# A New ‘Off–On’ System Based on Core‐Substituted Naphthalene Diimide with Dimethylamine for Reversible Acid–Base Sensing

**DOI:** 10.1002/open.202200060

**Published:** 2022-06-09

**Authors:** Vishal G. More, Dinesh N. Nadimetla, Geeta A. Zalmi, Vilas K. Gawade, Ratan W. Jadhav, Yogesh D. Mane, Sheshanath V. Bhosale

**Affiliations:** ^1^ School of Chemical Sciences Goa University Taleigao Plateau 403 206 Goa India; ^2^ Department of Chemistry BSS Art's, Science and Commerce College Makni Tq. Lohara 413604 Maharashtra Dist. Osmanabad India

**Keywords:** acid-base sensor, calorimetric, naphthalenediimide, reversible pH sensor, test strip

## Abstract

A new ‘Off–On’ system designed and synthesised by functionalisation of a naphthalene diimide (NDI) core with dimethylamine produces 4,9‐bis(dimethylamino)‐2,7‐dioctylbenzo[*lmn*][3,8]‐phenanthroline‐1,3,6,8‐(2*H*,7*H*)‐tetraone, abbreviated as DDPT (**1**). DDPT **1** was synthesised using a simple strategy, namely aromatic nucleophilic substitution using Br_2_‐NDI with dimethylamine at 110 °C. DDPT was characterized by ^1^H and ^13^C NMR spectroscopy, ESI mass spectrometry and elemental analysis. DDPT **1** was then used for optical studies through protonation of its dimethylamine core with trifluoroacetic acid (TFA), blue‐shifting the absorption band from 600 nm to 545 nm in solution. Interestingly, the fluorescence of DDPT **1** is weak in solution with a quantum yield Φ=0.09, which is significantly enhanced to Φ=0.78 upon addition of TFA. The limit of detection (LOD) was determined to 2.77 nm. Furthermore, DDPT **1** can be used for naked eyed detection not only under UV light (365 nm) but also using visible light, as clear changes can be clearly seen upon addition of TFA. The binding constant of DDPT was calculated to 2.1×10^−3^ m ^−1^. Importantly, DDPT **1** showed reversible switching by alternative addition of acid (TFA) and base (triethylamine) without loss of activity. Immobilised on paper, DDPT **1** can be used for strip‐test sensing in which the colour changes from blue to reddish when expose to TFA vapours and reverse in the presence of triethylamine vapours.

## Graphical abstract

**Core‐substituted naphthalene diimide** with dimethylamine groups acts as a colorimetric optical reversible pH sensor. The reversible nature of sensing with alternative addition of acid and base can be monitored by naked eye. The LOD of detection was found to be 2.77 nm with a fluorescence quantum yield 0.78. Thus, this derivatives may have practical applications in monitoring pH in the future.

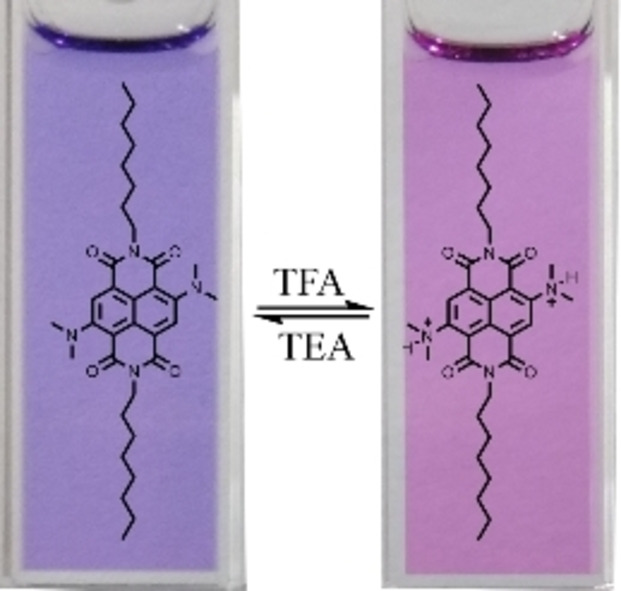

## Introduction

the pH value plays a critical role in many aspects such as chemical, environmental and biological processes. Hence, the accurate measurement of pH is of great significance in various fields of science and technology.^[1]^ The cell homeostasis regulation and various metabolic pathways are governed by pH, showing the importance to not only being limited to chemistry but also applying to biochemistry, cellular biology and drug delivery systems.[^2^, ^3^] To understand pH changes, different glass‐pH‐electrodes are widely used as pH sensors, measuring the pH in a wide range from acidic over neutral to basic media. The glass pH electrodes are free from interference, show low detection limit, and long‐term stability with excellent reproducibility.^[4]^ However, there are some drawbacks that limit the usage of these pH‐glass electrodes such as their temperature‐dependent nature, complex design and fragility. In addition, fabrication of the electrode is another difficult task, and their invasive nature makes it difficult to use them for more specialised sensing applications. Recently, optical pH sensing has thus attained great demand and became a widely accepted method as an alternative to glass‐pH electrodes.^[5]^

There are several fluorescent molecules that have been developed to be used for pH sensing applications which are typically based on absorption and fluorescence changes of the indicator. As they exhibit high sensitivity, they can be employed in different areas.^[6]^ There are different methods and devices available for measurement of pH, but fluorescence microscopy is among the most powerful tools for sensing applications. Fluorescent materials follow three important charge transfer mechanisms, namely photoinduced electron transfer (PET),^[7]^ internal charge transfer (ICT),^[8]^ and fluorescence resonance energy transfer (FRET).^[9]^ A chemosensor useful for PET consists of a fluorophore‐spacer‐receptor arrangement, comprising a PET acceptor (denoted as fluorophore) and a PET donor (an amine).^[10]^ Nevertheless, design, synthesis and application of the probe by applying the simplest synthetic strategies is very important. In addition, proper cell permeability is yet another necessary requirement for being able to detect pH in acidic cellular environments.^[11]^

Among planar aromatic molecules, naphthalene diimides (NDIs) have found wide application in various fields[^10^, ^12^, ^13^, ^14^, ^15^] due to their easy synthesis, that is, functionalisation at both the anhydride position and at the core. NDIs possess very good optical properties and strong fluorescence.^[12]^ Due to the planar nature of NDI, they have shown to be excellent candidates in the field of supramolecular chemistry. Various self‐assembled structures have been produced due to their flexible π–π‐interactions with solvophobic control. Interestingly, the optical properties of the NDI can be tuned by changing the core‐substitution of NDIs such as by introducing nitrogen, sulphur and oxygen.[^16^, ^17^, ^18^] Generally, NDI‐based molecules show non‐fluorescent behaviour in aqueous media due to the aggregation‐caused quenching phenomenon which is the most common process observed in many fluorophores. NDI furthermore possess π‐acidity, and together with their redox behaviour have thus attracted the attention of many researchers in various fields such as sensing, semiconductor materials, and self‐assembly applications.^[19]^ NDIs have better solubility, due to easy functionalization to the core, as compared to other organic molecules such as perylene, terrylene and quaterrylenes. Taking advantage of this. several NDI‐type core‐substituted aromatic amines have been synthesized for sensing applications.[^20^, ^21^, ^22^, ^23^, ^24^, ^25^] NDIs are especially more desirable in sensing application as they are small and can be easily functionalized with good solubility in different solvents and exhibit tuneable fluorescence based on the core substituents.

**Figure 1:**
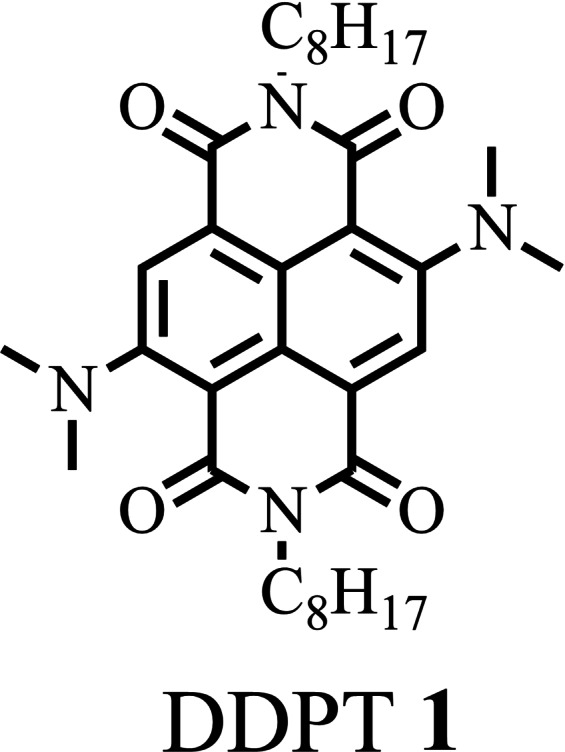
Chemical structure of DDPT **1**.

In this paper, we report a new core‐substituted NDI bearing dimethylamine substituents at both 2‐ and 6‐positions to produce 4,9‐bis(dimethylamino)‐2,7‐dioctylbenzo[*lmn*][3,8]‐phenanthroline‐1,3,6,8‐(2*H*,7*H*)‐tetraone (Abbreviated as DDPT **1**; shown in Figure 1) by reacting dibromo‐NDI with dimethylamine in the presence of *N*,*N’*‐dimethylformamide (DMF) and triethylamine (TEA) at 110 °C for 48 h.

The synthesised molecule was characterised by means of ^1^H and ^13^C NMR spectroscopy, ESI mass spectrometry and elemental analysis. DDPT **1** was further used for an acid‐base sensing application and shows ‘switch‐on’ emission upon addition of acid (TFA) which is reversed to the original state by addition of base (TEA). This phenomenon can be visualised by naked eye due to the changing in colour from blue to reddish, and vice versa. Furthermore, the DDPT **1** was not only used for calorimetric sensing but also for optical spectroscopy by means of UV‐Vis absorption and fluorescence spectroscopy. DDPT **1** can be used for strip‐based sensing, which may in future useful to replace litmus paper, as a strip functionalised with DDPT **1** can be reused several time.

## Results and Discussion

### Sensing Performance of DDPT 1

**Figure 2:**
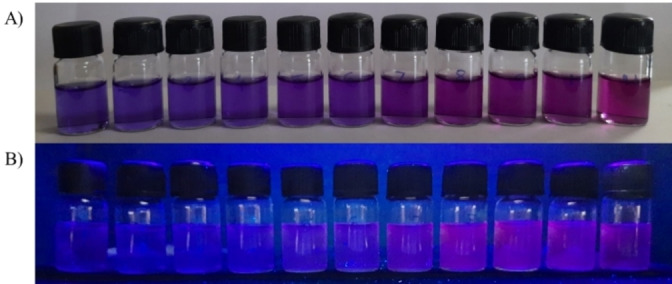
Sensing performance of the DDPT 1 (90 μm) in THF with the addition of trifluoroacetic acid (0.2 to 2 equiv.) A) under visible light B) under UV light illumination at 365 nm.

The sensing performance was studied for DDPT **1** (9×10^−5^ m) in THF upon addition of TFA (0–2.0 equiv.). The change in colour under day light and UV light (365 nm) were as illustrated in Figure 2. Under day light, DDPT **1** in THF shows blue colour which changes to a purple and then radish colour with incremental addition of TFA (Figure 2A). Under UV light (365 nm), DDPT **1** produces blue fluorescence colour upon incremental addition from 0–2 equiv. of TFA. The colour of the solution shows purple to red emission with increasing concentration of TFA. Thus the observed change in colour indicated that the DDPT **1** can be utilized for naked‐eye colorimetric and fluorescent sensing of H^+^ ions.

### UV‐Vis Absorption

**Figure 3:**
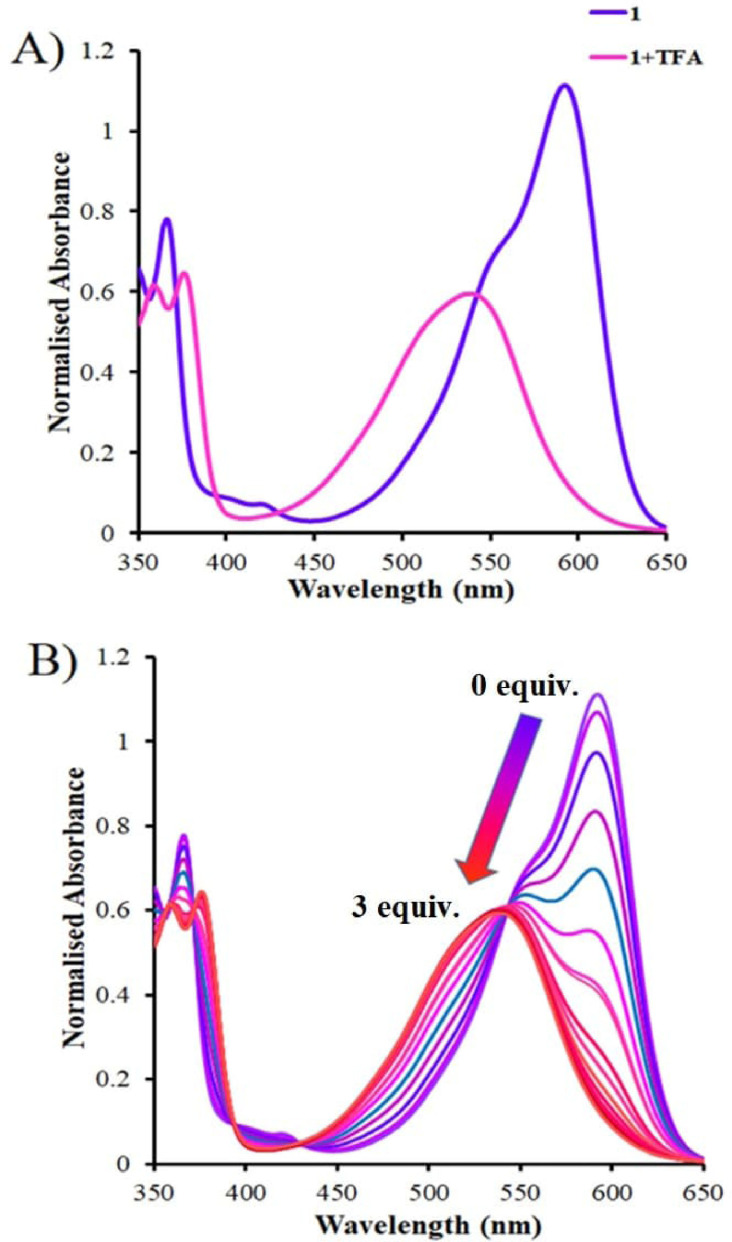
UV Vis absorption spectra of 1 (90 μm) and DDPT 1+TFA. B) Absorption spectra of DDPT 1 with incremental addition 0.2 equiv. of TFA started with 0 up to 3 equiv. in THF.

The UV‐Vis absorption spectrum for DDPT 1 (9×10^−5^ M) was recorded in THF, which typically produces a characteristic absorption band between 350 to 400 nm corresponding to the π–π^*^ transition of the NDI core. However, the broad and strong absorption band appeared at a longer wavelength band at 600 nm due to n–π^*^ transition, occurring because of the electron‐donating nature of dimethylamine group attached to the core of the NDI (Figure 3A). Thus, it is clear that the transition is mainly through donation of electron density to the NDI core from electron‐donating dimethylamino groups present on both substituted sides of the NDI core. Upon addition of TFA to the solution of DDPT 1, the absorption band at 600 nm is blue‐shifted to 545 nm and even more broadened (Figure 3A). The change in absorption is due to photoinduced electron transfer from quaternary amine to the core of NDI. This prominent change in absorption is due to protonation occurring at the tertiary amine on the NDI core to give the quaternary amine. However, the addition of TFA to the solution of NDI does not affect the absorption band appearing between 350 to 400 nm. The UV‐Vis changes in absorption ultimately suggest that the DDPT 1 is highly selective towards H^+^ ion.

In addition, UV‐Vis titration was performed for DDPT 1 as shown in Figure 3B. The absorption changes were recorded upon incremental addition of 0–2 equiv. TFA and it was observed that with increase in TFA concentration in the solution of DDPT 1, there is a shift in absorption with a frequency decrease of the absorption band at 600 nm and blue shifted band to 545 nm with isosbestic point appears at 550 nm.

### Fluorescence Emission

**Figure 4:**
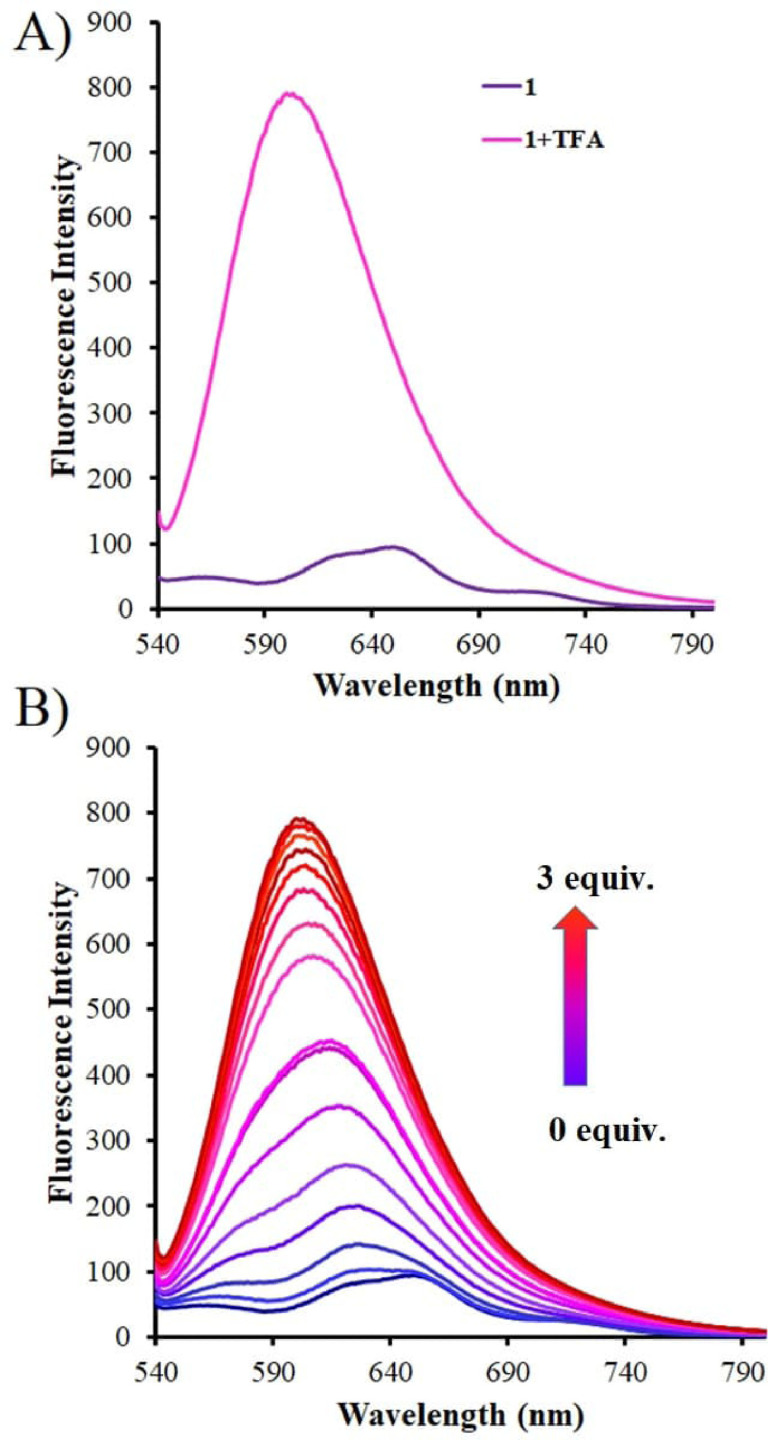
Emission spectra of DDPT 1 (90 μm) and 1+TFA. B) Emission spectra of DDPT 1 with incremental addition 0.2 equiv. of TFA started with 0 up to 3 equiv. (90 μm) in THF.

Moreover, a fluorescence emission study was performed for DDPT 1 towards the sensing of H^+^ ions in solution. The emission spectra of DDPT 1 were studied in various solvents as shown in Figure S9. Fluorescence results obtained are represented in Figure 4. It is observed that the DDPT 1, upon excitation at 520 nm, showed am emission band that appeared at 600 nm. The fluorescence colour change was observed upon addition of TFA from blue to purple. However, initially there was no significant change upon addition of TFA with weak emission intensity band at small concentrations. Upon incremental addition from 0–3.0 equiv. TFA, fluorescence emission enhancement was observed (Figure 4B), which is similar to colour changes from blue to purple as shown in Figure 2B under irradiation (λ=365 nm).

The fluorescence of native 1 was found to be very weak with a quantum yield in THF at room temperature only amounting to Φ=0.09, which is enhanced 9 times upon addition of 3 equiv. of TFA, that is, the quantum yield was increased by up to Φ=0.78. The choice of 3 equiv. of TFA is to see the saturation point in fluorescence.

### ^1^H NMR Titration with Addition of TFA

A ^1^H NMR titration experiment was carried out in order to study the effect on chemical shift upon protonation and deprotonation changes occurring in the molecule during addition of TFA in solution of the DDPT 1. It is observed in the ^1^H NMR titration that upon addition of 2 equiv. of TFA, new broad proton peaks appear (Figure S4, Supporting Information).

### Binding Constant

**Figure 5:**
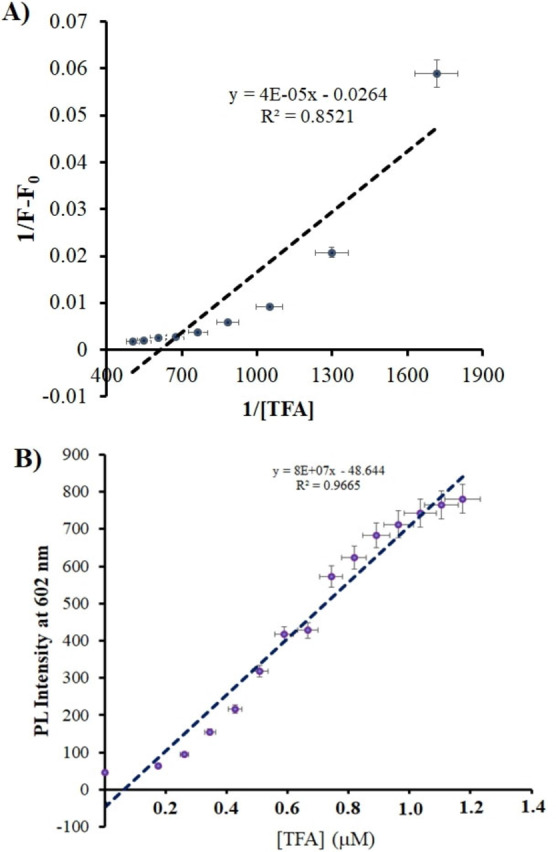
A) Benesi–Hildebrand plot, B) LOD plot of the DDPT 1.

The binding constant of DDPT 1 was further determined by using the Benesi–Hildebrand plot. The binding constant for the H^+^ to receptor was found to be 2.1×10^−3^ m ^−1^ (Figure 5A).

### Limit of Detection

The limit of detection can be calculated by LOD=3σ/S, where σ is the standard deviation of blank sample and S is the absolute value of the slope between absorption intensity and concentration of H^+^ ions. The limit of detection for the DDPT 1 is 2.77 nm (Figure 5B). This suggests that DDPT 1 could be employed as a sensitive fluorescent sensor for the quantitative detection of H^+^ ions. The recent literature on related pH sensors has been included in Table S1 for comparison.

### Reversibility and Reusability

A reversibility study was performed for DDPT 1 through alternative addition of TFA and TEA. It was observed that, upon addition of TFA, the protonation occurs at the secondary amine. Upon addition of 2 equiv. of triethylamine, DDPT 1 shows a reversal to the original state. Initially the DDPT 1 shows blue colour but addition of TFA changes the colour form blue to purple. However, on addition of base, the colour of the DDPT 1 from purple returns to the blue colour of the initial solution of the DDPT 1. The absorption and fluorescence spectra were recorded at room temperature for the DDPT 1 as shown in Figure S5 (Supporting Information). It is clearly observed that the addition of acid and base shows the reversible nature reproducing blue colour and shows the absorption close to the blank (Figure S8).

### Test Strip for Reversible Acid‐Base Sensing

**Figure 6:**
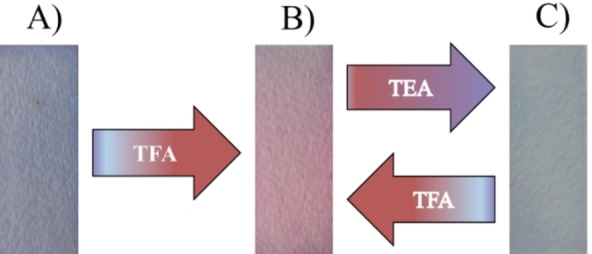
Test Strip sensing for DDPT 1 in THF solvent in presence of TFA and TEA. A) Test strip as blank. B) After exposed to TFA vapours. C) Further contact with TEA vapours.

Furthermore, we studied strip‐based sensing using DDPT 1 to show its practical applicability. The test strips were prepared by dissolving DDPT 1 in THF and simply loading the sample on the test strips made from Whatman paper. The test strips were air‐dried and used for further sensing application. The prepared strips initially showed blue colour in the visible light (Figure 6A) Upon subjecting the test strip to TFA fumes, the blue colour changes to light purple pink colour. In presence of base TEA, again, the colour of the test strip changes to blue, which indicates that the test strips show reversibility and reusability and can be used for practical application in pH sensing. The test strip has been utilized up to 8 cycles. (See Figure S7, Supporting Information)

## Conclusion

The probe 4,9‐bis(dimethylamino)‐2,7‐dioctylbenzo[*lmn*][3,8]‐phenanthroline‐1,3,6,8‐(2*H*,7*H*)‐tetraone (DDPT **1**) was synthesized with substituted dimethylamine at the core of NDI. The molecule was successfully synthesized and characterized showing the reversible response to trifluoroacetic acid which acts as proton source. The optical changes in the molecule showed the increase in fluorescence with the increase in proton source concentration. The reversible nature of the DDPT **1** was further investigated by adding TEA as base, acting as a deprotonator. This suggests that protonation of the N atom of the dimethylamino group restricts the PET transfer leading to an increase in fluorescence. The DDPT **1** was reversible for 8 cycles exposing a **1**‐loaded strip reversibly to acid and base. All results successfully indicate that DDPT **1** is highly selective to H^+^ ions in solution. We believe that this simple route for the synthesis of DDPT **1** and its use for reversible acid‐base sensing may be useful to monitor and control the pH in various biological and physiological processes.

## Experimental Section

### Chemicals and Reagents

**Scheme 1:**
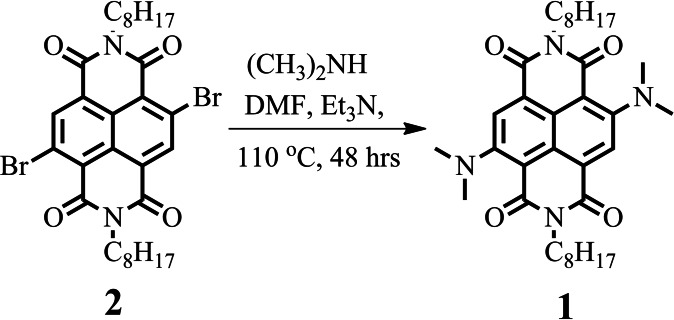
Synthesis of DDPT **1**.

Dibromo‐NDI was prepared by following our earlier developed method.^[7]^ DDPT 1 was synthesized by reacting dibromo‐NDI with dimethyl‐amine in presence of triethylamine as base in dry DMF as shown in the Scheme 1. Dimethylamine, trifluoroacetic acid and trimethylamine were purchased from Sigma‐Aldrich. ^1^H NMR spectra were recorded on Bruker spectrometer 400 MHz and ^13^C NMR using 100 MHz. The CDCl_3_ was used as a deuterated solvent for NMR with tetramethylsilane (TMS) was used as an internal standard. Mass spectrometry data was obtained by high resolution mass spectroscopy. IR spectra were recorded on a Perkin Elmer FTIR 400 spectrometer. UV‐Vis absorption spectra were recorded by UV‐Vis‐1800 Shimadzu spectrophotometer and fluorescence emission measured on RF‐6000 (Shimadzu, Japan) spectrofluorophotometer.

### Synthesis of DDPT 1

Synthesis of 4,9‐bis(dimethylamino)‐2,7‐dioctylbenzo[*lmn*][3,8]‐phenanthroline‐1,3,6,8‐(2*H*,7*H*)‐tetraone (DDPT **1**). The DDPT **1** was synthesized by reacting 100 mg of **2** in 5 mL of dimethylamine, 5 mL of triethylamine and 5 mL of dimethylformamide under N_2_ atmosphere by heating reaction mixture at 110 °C for 48 h. Upon completion of the reaction, mixture was extracted with ethyl acetate to give the desired product **1** as a violet blue compound in 75 % yield. ^1^H NMR (400 MHz, CDCl_3_) δ (ppm): 8.44 (1H, s), 4.19 (2H, *J*=7.5 Hz, t), 3.17 (6H, s), 1.75–1.67 (2H, m), 1.41–1.24 (10H, m), 0.87 (3H, *J*=6.8 Hz, t); ^13^C NMR (100 MHz, CDCl_3_) δ (ppm): 163.5, 161.8, 151.6, 125.2, 123.6, 123.2, 107.2, 44.1, 40.9, 31.9, 29.3, 29.2, 28.3, 27.1, 22.6, 14.1. HRMS: Calcd.=576.3676, found 576.3359; Elemental Analysis: Calcd. C, 70.80; H, 8.39; N, 9.71; found C, 70.68; H, 8.41; N, 9.78 %.

### UV‐Vis Absorption Spectra and Titration

The stock solution of DDPT **1** (9×10^−5^ m) was prepared by dissolving it in THF. Then absorption spectra were recorded by placing of stock solution in the quartz cuvette with 1 cm path length. The change in absorption upon addition of TFA was recorded at room temperature. Furthermore, changes in absorption was monitored in titration experiments, firstly DDPT **1** was placed in cuvette and upon addition of 0–3.0 equiv. of TFA.

### Fluorescence Emission Spectra of DDPT 1

The stock solution (9×10^−5^ m) was prepared by dissolving the DDPT **1** in THF. The fluorescence spectra of the DDPT **1** was recorded by placing of stock solution in THF in the quartz cell. The fluorescence change upon addition of TFA was recorded at room temperature. And for titration experiment 0–3.0 equiv. TFA was added incrementally and the change in fluorescence spectra. The excess TFA was used in FL study, to see saturation point.

## Conflict of interest

The authors declare no conflict of interest.

1

## Supporting information

As a service to our authors and readers, this journal provides supporting information supplied by the authors. Such materials are peer reviewed and may be re‐organized for online delivery, but are not copy‐edited or typeset. Technical support issues arising from supporting information (other than missing files) should be addressed to the authors.

Supporting Information

## Data Availability

The data that support the findings of this study are available in the supplementary material of this article.
