# Supplementary material for: A New ‘Off–On’ System Based on Core‐Substituted Naphthalene Diimide with Dimethylamine for Reversible Acid–Base Sensing
Source: ChemistryOpen. 2022 Jun 9;11(6):e202200060. doi: 10.1002/open.202200060 (PMC9179010; doi:10.1002/open.202200060)
Supplement: Supplementary file 1 — Supporting Information [file OPEN-11-e202200060-s001.pdf]

ChemistryOpen

Supporting Information

A New ‘Off–On’ System Based on Core-Substituted Naphthalene Diimide with Dimethylamine for Reversible Acid–Base Sensing

Vishal G. More, Dinesh N. Nadimetla, Geeta A. Zalmi, Vilas K. Gawade, Ratan W. Jadhav, Yogesh D. Mane,* and Sheshanath V. Bhosale*

SUPLLEMENTARY INFORMATION

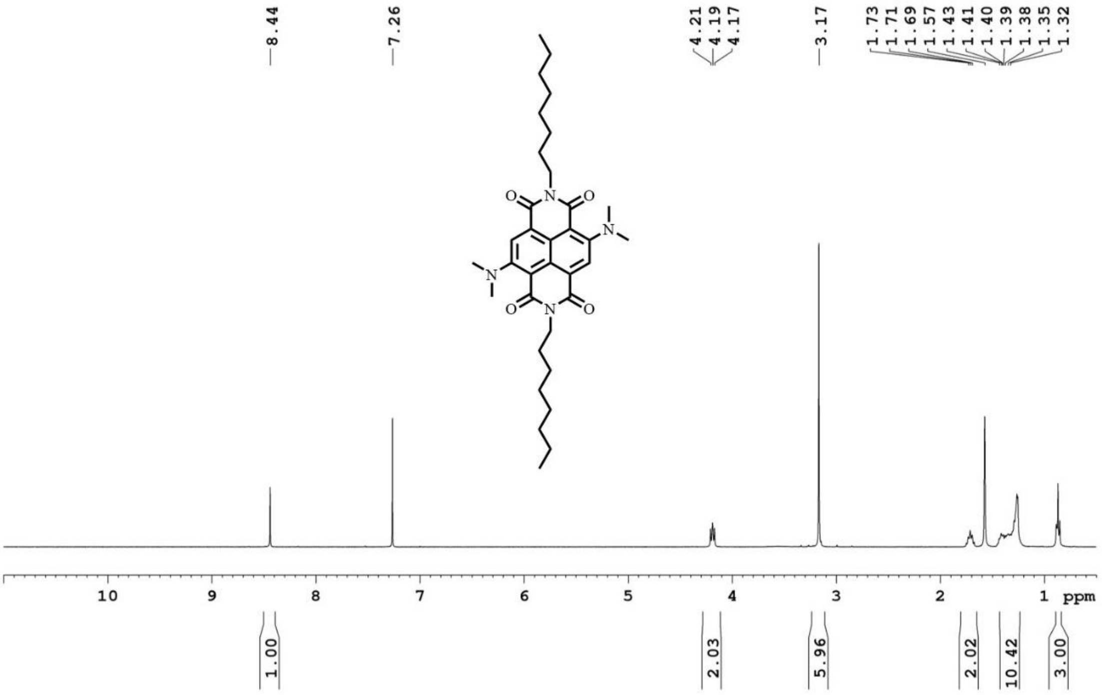

Figure S1. ^{1}H NMR spectra of the compound 4,9-bis(dimethylamino)-2,7-dioctylbenzo[lmn][3,8]phenanthroline-1,3,6,8(2H,7H)-tetraone **(**DDPT **1**).

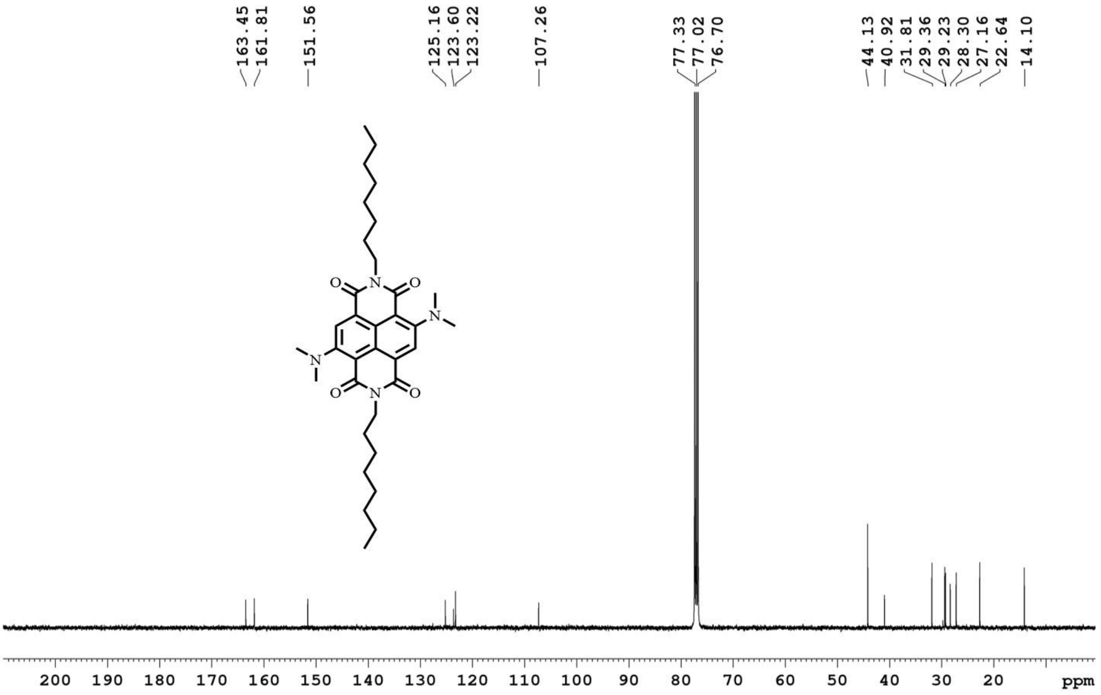

Figure S2. ^{13}C NMR spectra of the probe 4,9-bis(dimethylamino)-2,7-dioctylbenzo[lmn][3,8]phenanthroline-1,3,6,8(2H,7H)-tetraone (DDPT **1**).

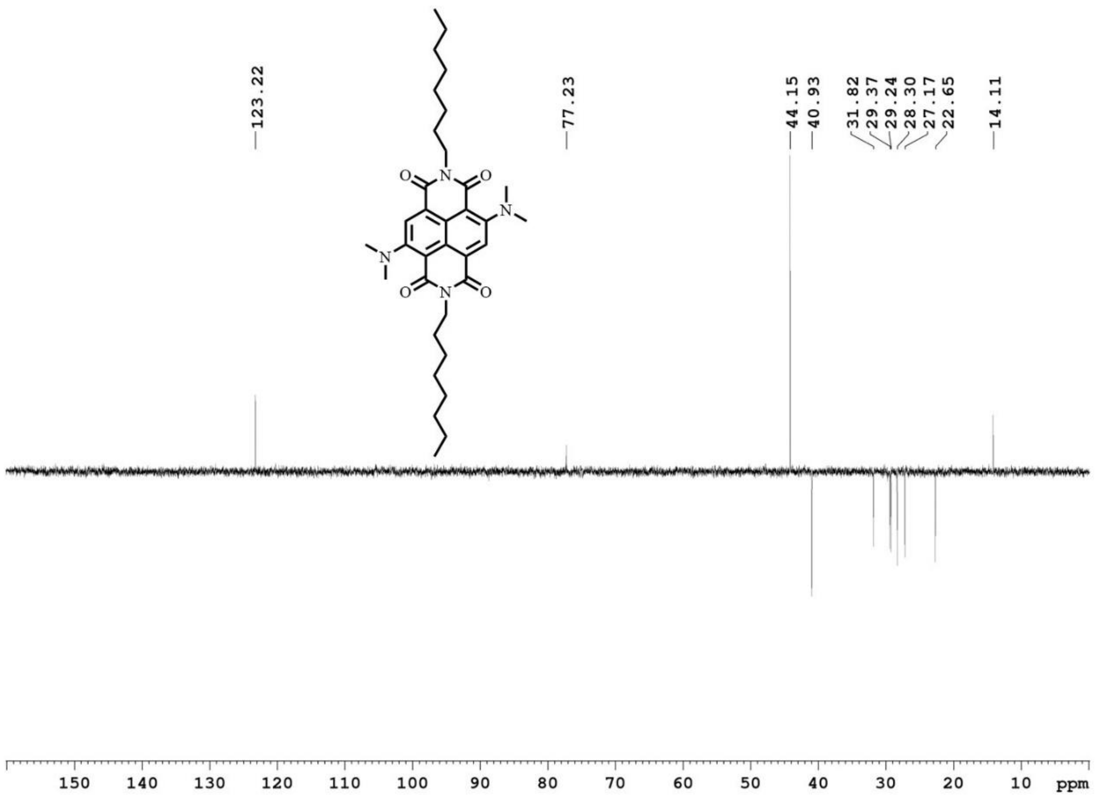

Figure S3. DEPT spectra of the probe 4,9-bis(dimethylamino)-2,7-dioctylbenzo[lmn][3,8]phenanthroline-1,3,6,8(2H,7H)-tetraone (DDPT **1**).

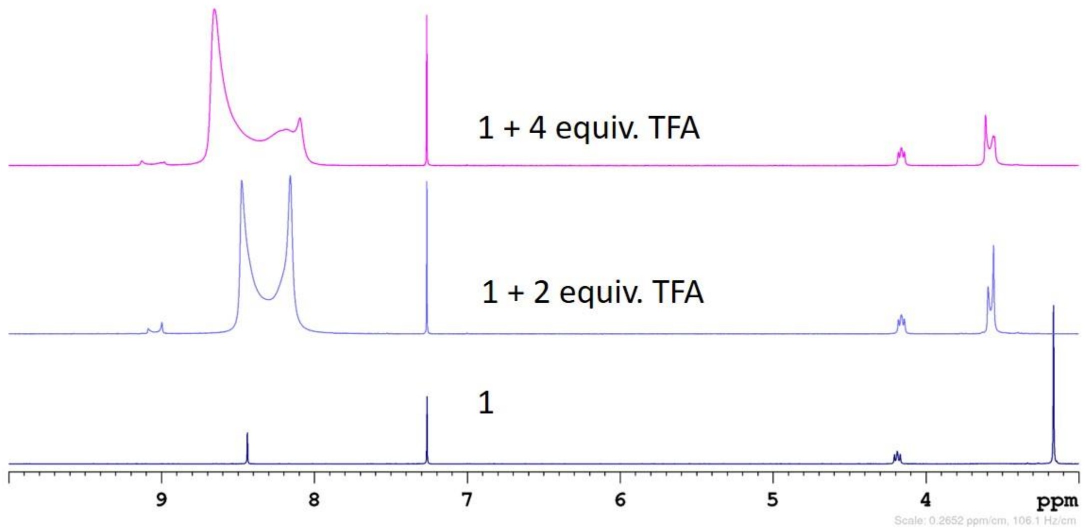

Figure S4. ^{1}H NMR titration of the probe 4,9-bis(dimethylamino)-2,7-dioctylbenzo[lmn][3,8]phenanthroline-1,3,6,8(2H,7H)-tetraone (DDPT **1**).

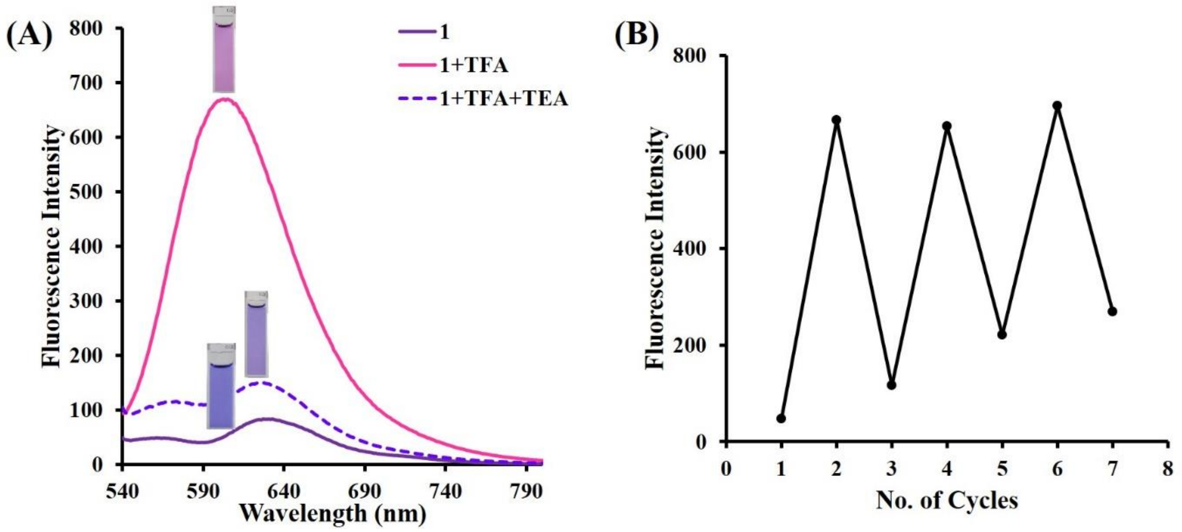

Figure S5. Reversibility study of the probe 4,9-bis(dimethylamino)-2,7-dioctylbenzo[lmn][3,8]phenanthroline-1,3,6,8(2H,7H)-tetraone DDPT (A) representing the fluorescence intensity changes occurring upon addition of TFA to the DDPT **1** (B) fluorescence intensity representing the number of reversible cycle for the DDPT **1**.

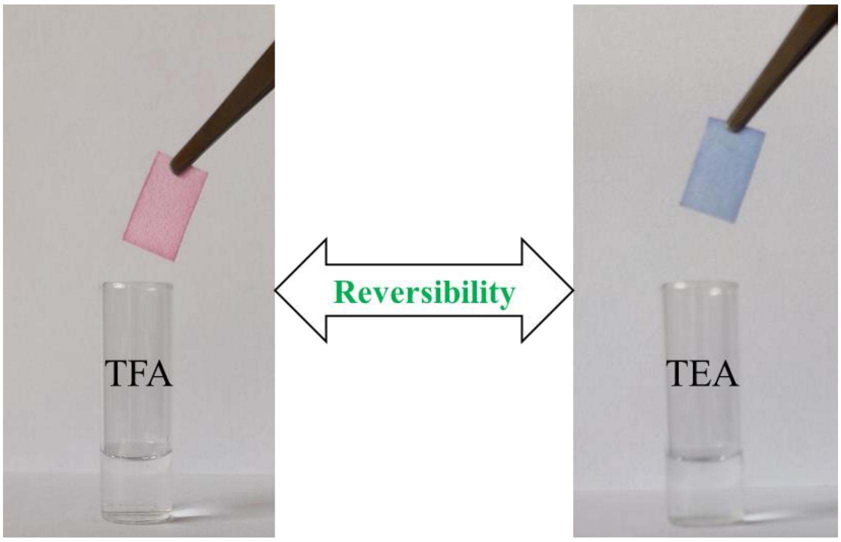

Figure S6. Photograph of test strips demonstrating the color change upon subjecting the strip on TFA and TEA fumes.

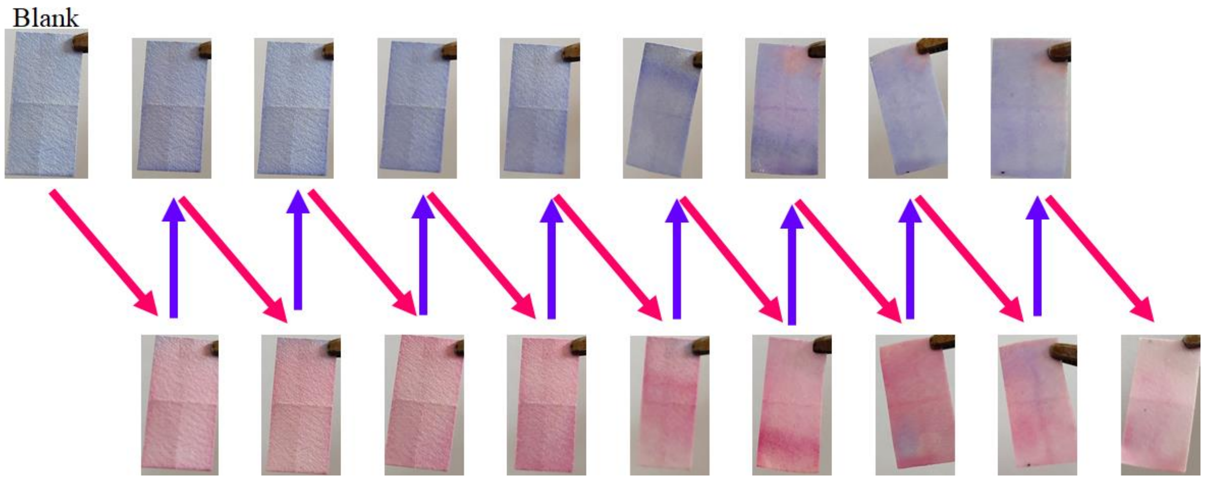

Figure S7. Reusability of test strip of DDPT **1** shows up to 8 cycles. (pink arrow and purple arrow indicates exposure to TFA and TEA fumes respectively).

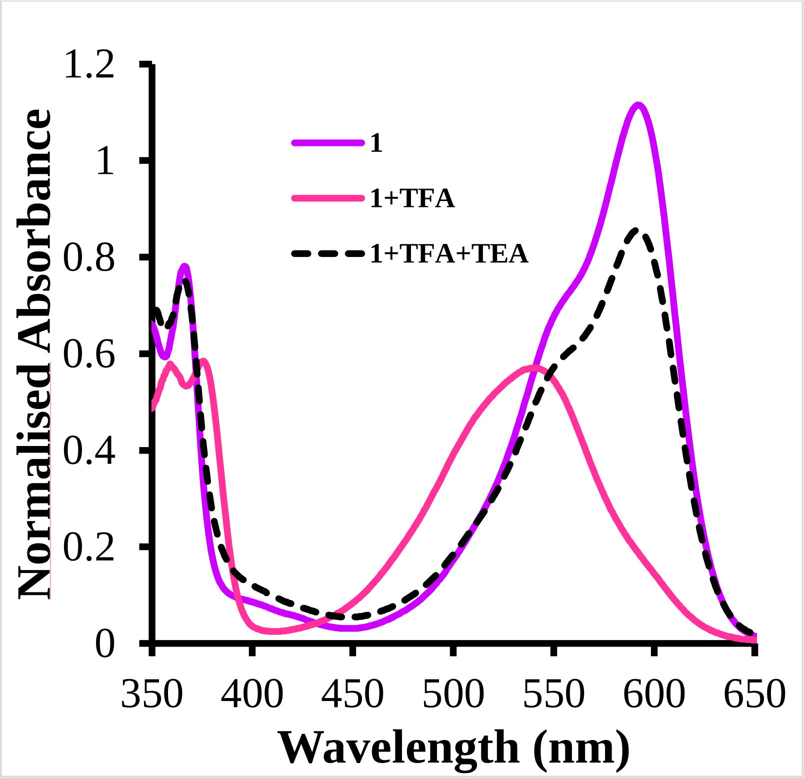

Figure S8. Reversibility study of the DDPT **1** the (purple color curve) represent DDPT **1** upon addition of acid i.e. TFA it gives (pink color curve) and its shown (black dotted curve) which shows reversibility while upon addition of base i.e. TEA.

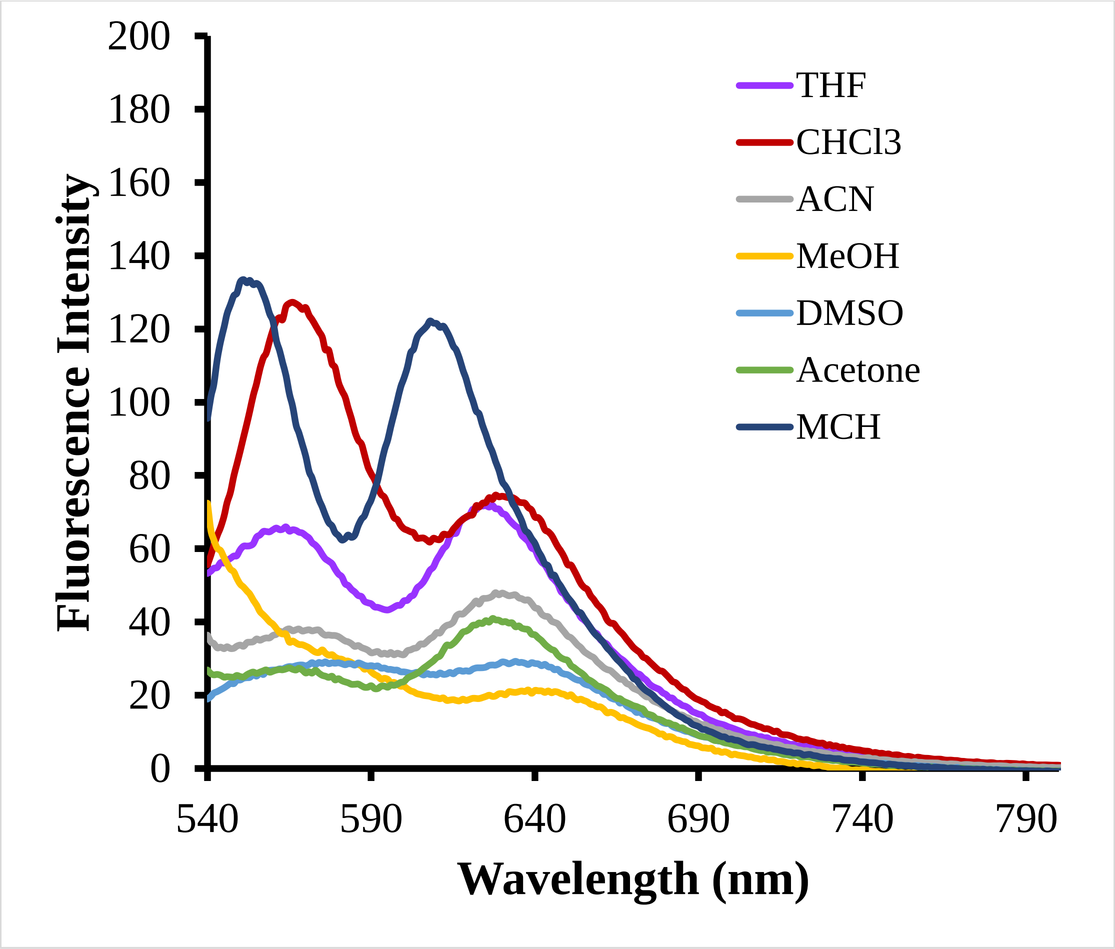

Figure S9. Emission spectra of DDPT **1** in various organic solvent.

Table S1. Comparison of pH sensing with recent literature

Sr.No	Compound	Sensing method	LOD	Test strip	Reversibility	References
1	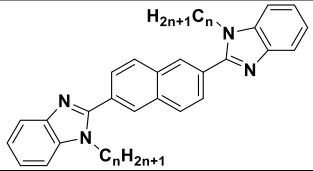	Fluorescence	-	No	Yes	[1]
2	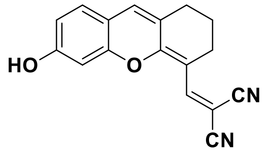	Fluorescence	-	No	yes	[2]
3	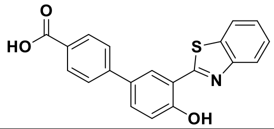	Ratiometric fluorescence	-	Yes	Yes	[3]
4	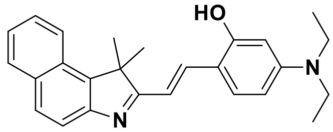	Fluorescence Turn ON	2.967μM	No	No	[4]
5	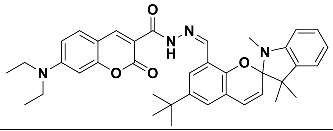	Colorimetric and fluorescence	-	No	yes	[5]
6	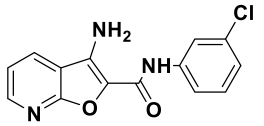	Fluorescence	-	No	No	[6]
7	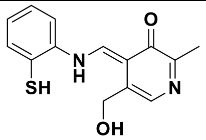	Fluorescence	-	No	No	[7]
8	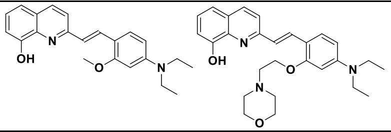	Fluorescent	-	No	No	[8]
9	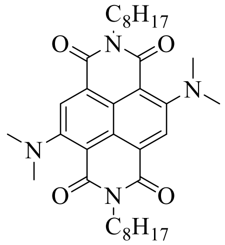	Naked eye colorimetric fluorescent “turn ON” receptor for pH sensing	2.77 nM	Yes	Yes	This work

References

[1] Y. C. Wu, J. Y. You, K. Jiang, H. Q. Wu, J. F. Xiong, Z. Y. Wang, *Dye. Pigment.* **2018**, *149*, 1–7.

[2] M. Liu, Y. Lv, X. Jie, Z. Meng, X. Wang, J. Huang, A. Peng, Z. Tian, *Sensors Actuators, B Chem.* **2018**, *273*, 167–175.

[3] K. Li, Q. Feng, G. Niu, W. Zhang, Y. Li, M. Kang, K. Xu, J. He, H. Hou, B. Z. Tang, *ACS Sensors* **2018**, *3*, 920–928.

[4] Y. Zhang, Y. Zhao, A. Zhou, Q. Qu, X. Zhang, B. Song, K. Liu, R. Xiong, C. Huang, *Spectrochim. Acta - Part A Mol. Biomol. Spectrosc.* **2021**, *261*, 120014.

[5] X. He, W. Xu, C. Xu, F. Ding, H. Chen, J. Shen, *Dye. Pigment.* **2020**, *180*, 108497.

[6] L. Zhang, Y. Liu, X. Li, Y. Guo, Z. Jiang, T. Jiao, J. Yang, *ACS Omega* **2021**, *6*, 4800–4806.

[7] K. Bamnavat, V. Bhardwaj, T. Anand, S. A. Kumar, S. K. Sahoo, *Dye. Pigment.* **2021**, *184*, 108844.

[8] S. Guria, A. Ghosh, T. Mishra, M. kumar Das, A. Adhikary, S. Adhikari, *J. Photochem. Photobiol. A Chem.* **2021**, *407*, 113074.
